# How will the ebola crisis impact the HIV epidemic?

**DOI:** 10.1186/s12977-014-0110-z

**Published:** 2014-11-30

**Authors:** Mark A Wainberg, Andrew M L Lever

**Affiliations:** McGill University AIDS Centre, Jewish General Hospital, Montreal, QC Canada; Department of Medicine, University of Cambridge, Cambridge, UK

The dramatic spread of the Ebola virus in countries in Western Africa over the past year has wreaked havoc throughout that region and brought panic and fear to millions of people around the world. There are many similarities between people’s attitudes today toward Ebola virus and its victims and the attitudes to HIV that were prevalent only 25 years ago. In the run-up to World AIDS Day, December 1, we should reflect on these parallels between HIV and Ebola virus and what may be the potential impact of the Ebola crisis on efforts to control the spread of HIV in the countries that have been so badly affected by both of these lethal pathogens.

Over 39 million people have already died of HIV disease and it is estimated that 35 million individuals are currently infected by the virus. Although the estimated number of cases of Ebola is still comparatively small – around 17,000 - there is, understandably, widespread fear that Ebola transmission may intensify. Moreover, a tragically high proportion of Ebola-infected individuals, possibly 5,000 - 6,000, have died of their disease. Although several healthcare volunteers from developed countries who contracted Ebola while working in Western Africa have died since returning home, other westerners have survived due, in large part, to the outstanding treatment that they have received at tertiary care hospitals in their home countries. As today for HIV, this provides testimony to the far worse prognosis for severe infections in settings in which appropriate health care is not available. Again, as for HIV, it underlines the importance of the recent initiatives to set up better health care provision in Ebola affected countries.

Publicity about Ebola has eclipsed that of HIV, but it is salutary to remember that approximately 10 times fewer people die each day in Africa of Ebola than of HIV/AIDS. However, what is certain is that the Ebola crisis will have a major effect on HIV/AIDS (and vice versa) in the countries in which this new epidemic has taken hold. In part, this is because a disproportionate percentage of those infected by Ebola have themselves been health care professionals, which must have negative consequences for the care of persons suffering from other diseases including HIV. Suboptimal treatment and control of HIV in these areas equates to a population more susceptible to Ebola, and the understandable shift in attention to Ebola potentially compromises HIV control measures such as HIV testing, HIV treatment, treatment as prevention, and prevention of mother to child transmission. Furthermore, accessing HIV clinics in parts of Western Africa may now be compromised by fear of Ebola and by new constraints on public transportation. Despite the fact that wealthy countries contribute billions of dollars annually toward global HIV drug purchase programmes, the Ebola crisis has further strained the reservoirs of human resources in affected areas.

There are parallels in public perceptions of these two viruses. The panic that Ebola has evoked in Western Africa - and the West, following repatriation of Ebola-infected individuals - is reminiscent of attitudes toward HIV in the 1980s when people of Haitian origin, those individuals with hemophilia infected through blood products, and the gay population were discriminated against because they were thought to represent a risk to others for HIV acquisition.

This same stigma is now attaching to those courageous individuals who have volunteered to provide care to Ebola-infected individuals in Western Africa, people who should deserve our deep gratitude. These same attitudes were prevalent at the start of the AIDS epidemic when the belief existed that contact with health care professionals caring for AIDS patients, or with scientists working with HIV, was a risk for HIV acquisition. Unfortunately, groundless fears have never prevented unfounded hostile and discriminatory public attitudes.

Today, antiretroviral drugs (ARVs) provide hope to millions of HIV-infected individuals for a fully productive life and an extended, perhaps normal, lifespan. This progress has required many years of basic biomedical, clinical, and social research that has also resulted in dramatic improvements in the potency and tolerability of the ARVs that are used in therapy. Ebola, by contrast, despite being identified in 1976, before HIV, has enjoyed comparatively little research input. The perceived threat to Western nations has, as for HIV, triggered new efforts for Ebola drug discovery and vaccine development, but research on Ebola is still badly lagging behind. This situation can only be remedied by providing the necessary financial support and by providing social assistance for Ebola-infected individuals and their families and caregivers. Such research, in the case of HIV, not only improved treatment, but assisted in mitigating the problems of HIV discrimination and stigmatization as well as providing an understanding of HIV transmission.

Community engagement will be essential for Ebola, as was the case for HIV, and implementation of community awareness sessions and teaching programmes will be needed if we are to achieve the behavioral and socio-cultural changes required for elimination of stigmatization of Ebola-exposed persons. Both the Ebola and HIV epidemics highlight the need to strengthen health care systems in resource-limited settings as well as illustrating the sorts of measures that will limit Ebola transmission.

Ebola causes an acute disease that often leads to death rather than a chronic illness; thus, the window for transmission is far less than for HIV, meaning that it should theoretically be easier to expeditiously end the Ebola epidemic compared to HIV. However, the lessons learned from HIV must be applied to Ebola as quickly as possible.

Hopefully, new research into vaccines and antiviral drugs, together with public health control measures, will quickly lead to an end to the Ebola epidemic and restore normality to the fragile under-resourced societies that have suffered the most from Ebola. Perhaps the provision of new health care facilities now being set up in West Africa will provide support to their infrastructure in the future, when hopefully Ebola is controlled, and will aid them in efforts to raise standards of health care generally. It may even provide new pointers to yet further progress in the battle against HIV.

